# Evolution and Functional Divergence of the *Fructokinase* Gene Family in *Populus*

**DOI:** 10.3389/fpls.2020.00484

**Published:** 2020-05-14

**Authors:** Weijie Xu, Yiyang Zhao, Sisi Chen, Jianbo Xie, Deqiang Zhang

**Affiliations:** ^1^Beijing Advanced Innovation Center for Tree Breeding by Molecular Design, College of Biological Sciences and Technology, Beijing Forestry University, Beijing, China; ^2^National Engineering Laboratory for Tree Breeding, College of Biological Sciences and Technology, Beijing Forestry University, Beijing, China; ^3^Key Laboratory of Genetics and Breeding in Forest Trees and Ornamental Plants, College of Biological Sciences and Technology, Beijing Forestry University, Ministry of Education, Beijing, China

**Keywords:** FRK gene family, lineage-specific gene, adaptation, functional novelty, *Populus tomentosa*

## Abstract

New kinase has emerged throughout evolution, but how new kinase evolve while maintaining their functions and acquiring new functions remains unclear. Fructokinase (FRK), the gateway kinase to fructose metabolism, plays essential roles in plant development, and stress tolerance. Here, we explored the evolution of *FRK* gene family in 20 plant species (from green algae to angiosperms) and their functional roles in *Populus*. We identified 125 putative *FRK* genes in the 20 plant species with an average of 6 members per species. Phylogenetic analysis separated these 125 genes into 8 clades including 3 conserved clades and 5 specific clades, the 5 of which only exist in green algae or angiosperms. Evolutionary analysis revealed that *FRK* genes in ancient land plants have the largest number of functional domains with the longest amino acid sequences, and the length of *FRK* genes became shorter during the transition to vascular plants. This was accompanied by loss, acquisition, and diversification of functional domains. In *Populus*, segmental duplication appears to be the main mechanism for the expansion of *FRK* genes. Specially, most *FRK* genes duplicated in salicoids are regulated by *Populus*-specific microRNAs. Furthermore, compared with common *FRKs, Populus*-specific *FRK*s have showed higher expression specificity and are associated with fewer growth and wood property traits, which suggests that these *FRKs* may have undergone functional divergence. Our study explores the specific roles of *FRKs* in the *Populus* genome and provides new insights for functional investigation of this gene family.

## Introduction

Evolution is accompanied by various changes including morphological, physiological and genome, which allow organisms to deal with challenging conditions, including increased CO_2_ concentration, desiccation, changes in light intensity, high temperature, marked seasonal changes, and limited nutrient availability (Delaux et al., 2012; Romani et al., 2018). At the cellular level, changes of these physiological are regulated by kinase, which are major drivers of evolution (Michelson et al., 1985; Mehlgarten et al., 2018). New kinase classes evolved to orchestrate the complex development and environmental responses of higher organisms. Meanwhile, the birth, loss, and the expansion of kinase families has been demonstrated in each fully sequenced eukaryote genome (Mehlgarten et al., 2018).

As an important kinase to catalyze the key metabolic step of fructose phosphorylation, fructokinase (FRK) helps phosphorylates fructose to form fructose 6-phosphate (F6P) and functions in all stages of plant development (Plaxton, 1996). In addition, the *FRK* gene family is characterized by the presence of a phosphofructokinase-B (PfkB) domain and exists in all living organism. The PfkB domain possesses two notable motifs: a di-GLY (GG) motif in the N-terminal region and a GXGD or AXGD motif in the C-terminal region (Mcmahan et al., 1997; Kuettel et al., 2011). The function of each motif has been identified by structural and mutational analyses. For example, the aspartate in the GXGD or AXGD motif acts as a factor during catalysis, and it activates the C6 fructose hydroxyl group for nucleophilic attack on the γ-phosphate in ATP; the GG motif which connects the lid supplies flexibility in the hinge region (Riggs et al., 2017).

The evolution of *FRK* gene families across the plant kingdom has not been extensively studied. A phylogenetic analysis clustered *FRK* genes from dicotyledons and monocotyledons into six clades (Chen et al., 2017) and *FRK* genes from angiosperms into three clades (Riggs et al., 2017). In the moss *Physcomitrella patens*, *FRK* genes are clustered into two clades (Stein and Granot, 2018). These observations may be biased because of a limited number of organisms used in the studies. Therefore, the evolutionary history and origin of *FRKs* remains to be fully investigated. Furthermore, the availability of complete annotations of several plant genomes helped us to embark on a comprehensive evolutionary study of *FRKs*.

By changing in the native expression levels of *FRK* genes in transgenic plants through knock-down, knock-out and ectopic expression, *FRK* genes of different clades may have different or divergent physiological charactercharacters in angiosperm species, which can induce different phenotypes (Kanayama et al., 1997). For example, the growth of different tomato (*Solanum lycopersicum*) *FRK* knock-down lines was affected to different degrees, as knock-down *FRK2* affected more plant size than knock-down *FRK1* (Saori et al., 2002; Stein et al., 2017). *FRK2* is involved in xylem and phloem development, and RNA interference RNAi-mediated knock-down of tomato *FRK3* resulted in reduced water conductance (Saori et al., 2002). Simultaneous knock-down of both *FRK2* and *FRK3* led to huge and severe defects in plant growth and development, and it was more acute than those seen in *FRK2* knock-down plants alone (Stein and Granot, 2018). The diverse roles of *FRK* members suggest that the functions of these members in *FRK* family may be diverged.

*Populus* is a genus of woody perennial plants, currently it represents the most important and available tree model system for plant genomics (Tuskan et al., 2006). Throughout evolution, *Populus* has undergone three whole-genome duplication events (Tuskan et al., 2006), leading to the increase of genes which may significantly associate with lignocellulosic wall biosynthesis, disease resistance and metabolite transport (Tuskan et al., 2006). Trees have a long generation time but are short of characterized mutants, which impedes potential functional analysis of genes or gene families (Shanfa et al., 2013). However, single nucleotide polymorphism (SNP) based on the association mapping analysis has emerged as an effective and reliable way to explore the effect of genes on variation of phenotypic in perennial woody plants (Thumma et al., 2005). For example, in the previous studies of candidate gene-based association, several pivotal SNPs within candidate genes which associated with tree wood property and growth traits in *Populus* and *E. nitens* have been identified (Thumma et al., 2005).

In this work, we mainly focused on the evolution and functional divergence of the *FRK* gene family and the generation of functional novelty of new *FRK* gene. We strictly performed an analysis of phylogenetic of the *FRK* genes in diverse plant species and analyzed the predicted gene structures, motif composition and auxiliary domains of the *FRKs*, aiming to explore the functional divergence of this gene family in evolution. What’s more, these auxiliary domains will help us to track and analyze the evolutionary process of the *FRK* gene family, including gene loss and gene duplication events; and help us to associate these domains with the functional role of each class of *FRK* genes. Finally, we profiled the expression of *FRKs* in *P. tomentosa* and examined their genetic effects by association analysis. Our results help people to have a better understanding of evolution of lineage-specific *FRK* gene and provide a framework for the study of the functional divergence of gene families.

## Materials and Methods

### Identification of the *FRK* Genes in Viridiplantae

High-confidence *FRK* genes of *Arabidopsis thaliana*, *FRK1–FRK7* (Riggs et al., 2017) were used as reference sequences to identify *FRK* genes from Phytozome v11.0^1^ using a BLASTp search (*E*-value < 0.01) (Zaykin et al., 2002). Amino acid sequences, in which we identified a PfkB domain (PF00294) by using the PFAM^2^ database, were considered as putative members of *FRK* gene. Subsequently, we calculated the protein length, molecular mass, and theoretical pIs of FRKs in 20 plant species using ExPASy^3^ (Gasteiger et al., 2005).

### Amino Acid Sequence Alignment of FRKs and Phylogenetic Analysis

We performed phylogenetic analyses of the *FRK* gene family from 20 plant species: six chlorophytes: *Chlamydomonas reinhardtii*, *Dunaliella salina*, *Volvox carteri*, *Coccomyxa subellipsoidea*, *Micromonas pusill*, and *Micromonas* sp. *RCC299*; two embryophytes: *Marchantia polymorph*a and *P. patens*; one lycophyte: *Selaginella moellendorffii*; one gymnosperms: *Picea abies*; one basal angiosperm: *Amborella trichopoda*; eight Eudicot: *Brachypodium distachyon*, *Brassica napus, A*. *thaliana*, *S. lycopersicum, Eutrema salsugineum*, *Vitis vinifera, Eucalyptus grandis* and *Populus trichocarpa*; and two monocots: *Zea mays* and *Oryza sativa*. We used MAFFT software with default settings to perform multiple sequence alignments (Szerypo et al., 2003), after which alignments were manually adjusted in BioEdit software (Tippmann, 2004). Phylogenetic trees were constructed using the maximum likelihood (ML) method implemented in RAxML (Silvestro and Michalak, 2012) software; branch support was estimated with 100 bootstrap replicates.

### Inference of the Gain and Loss of *FRK* Clades

Under the Dollo parsimony rule, the gain and loss of different clades of *FRK* families, as well as the ancestral state, were assessed by using the COUNT software (Csűös, 2010) based on the presence or absent of *FRK* clades.

### Gene Structure and Domain Analyses

To better display the characteristics of *FRK* gene structure, genomic sequences were downloaded from Phytozome.11 database, and the conserved motifs and domains of the full-length amino acid sequences of FRKs were identified using the Multiple Expectation Maximization for Motif Elicitation (MEME)^4^ database and Pfam database. Relative parameters used in the analysis were as follow: the maximum number of motifs was 10 and the optimum width of motifs was set from 15 to 50. Furthermore, all identified motifs were annotated according to InterProScan database^5^ (Zdobnov and Apweiler, 2001). The structures of *FRK* genes were examined by the GSDS^6^ based on the genomic sequence and coding sequence of each *FRK* gene (Hu et al., 2014).

### Identification of *FRK* Genes Which Originated From Different Duplication Mechanisms

By using the methods of a previous study (Yun et al., 2011), *FRK* genes originated from tandem duplication or inverted duplication in *Populus tomentosa* were identified. *FRK* genes originated from segmental duplication were detected by using McscanX software (Yupeng et al., 2012).

### Identification of Potential miRNAs Regulating *FRK* Genes in *Populus tomentosa*

Regulatory mechanisms and the potential target site of miRNAs were predicted by using PsRNATarget^7^ based on 401 published miRNA sequences in *P. trichocarpa* (Puzey et al., 2012). Parameters were set as follows: maximum expectation score, 3; penalty for other mismatches, 1.0 (Dai et al., 2018). *Populus*-specific miRNAs were identified in a previous study (Xie et al., 2017). To further understand the potential regulation site of the *FRK* genes in *Populus*, we used MEME v.4.10.2^4^ in zoop mode (zero or one occurrence per sequence) to predict all conserved sequences in the transcripts. The parameters were set as follows: maximum number of motifs, 5; maximum and minimum motif width, 50 and 6, respectively; and default value for other parameters (Bailey et al., 2006).

### Reverse Transcription Quantitative PCR Analysis

All RNA was isolated from the mature leaf, immature leaf, elongated leaf, petiole, immature xylem, mature xylem, cambium, phloem, apex, and root of a 4-year-old *Populus tomentosa* clone “LM50” which planted in Guan Xian Country, by using the Plant RNA Kit (Magen, China). For biological replicates, this study used three individuals from one genotype of *P. tomentosa.* RNA was collected from third to eighth leaves of 1-year-old *P. tomentosa* clone “LM50,” and it was treated with the following stress treatments (three biological replicates per treatment): Drought stress was simulated by osmotic stress from treatment with 30% polyethylene glycol (PEG) solution. For the high temperature treatment, the seedlings were maintained at 42°C. The abscisic acid (ABA) treatment was applied by growing the seedlings on media supplemented with 100 mM/L ABA. The heavy metal treatment used lead (Pb) and was applied by growing the seedlings on a Pb (lead salt) 200 mg/L solution. The plants were treated with all stresses for 0, 1, 3, 6, and 12 h, after which leaf tissues samples were collected, frozen immediately in liquid nitrogen, and stored at 980°C for RNA isolation.

Primers were designed according to the corresponding *FRK* genes’ sequences from *P. trichocarpa* genome. RNA samples were treated with DNase I (TaKaRa, Beijing, China), and then the PrimeScript RT reagent Kit (Perfect Real Time) with gDNA Eraser (Takara, Beijing, China) was used for cDNA synthesis with oligo (dT) as the primer. The 7500 Fast Real-Time PCR System (Applied BIOsystems, Foster City, CA, United States) and the SYBR Green Premix Ex TaqTM (TaKaRa, Beijing, China) were used for all the reverse transcription quantitative PCR (RT-qPCR) assays. All the PCRs were performed using the two-step RT-qPCR procedure, and three replications per sample were performed to reduce the experimental error. We plotted a heat map of the transcription level of *FRK* genes across different tissues by using the pheatmap package in R. The genes and corresponding primers are listed in Supplementary Table S1.

### Measurement of Tissue Specificity

To analyze the tissue specificity of *FRK* gene expression, the tissue specificity score (Liao and Zhang, 2006) was computed as follows: With a_*ij*_ indicating the average expression level of gene *i* in tissue *j*, and the tissue specificity of gene *i* is defined by:

$$T\,\text{i}=\frac{1}{\text{n}-1}\,\sum_{j=1}^{\text{n}}\left(1-\frac{a_{i\,j}}{\left(a_{i\,j}\right)\,\max_{j}}\right)$$

With n indicating the number of tissues we used. Therefore, when the expression of a gene is the same in all tissues we used, the score is 0; when a gene is expressed in just one tissue, the score is 1.

### Population and Phenotypic Data

Correlative association population used in this study was composed of 435 unrelated *Populus tomentosa* individuals from a collection in Guan Xian town, Shandong province (China, 36°23′N, 115°47′E), covering nearly all the natural distribution individuals of *Populus tomentosa* species, including the northwestern, southern, and northeastern regions of China (30–40°N, 10–125°E) (Du et al., 2012). These 435 individuals were used to discover single nucleotide polymorphism (SNP)s within candidate *FRK* genes.

We used three growth traits and seven wood property traits for the association analysis. The seven wood properties were as follow: holocellulose content (HC, %), lignin content (LC, %), α-cellulose content (CC, %), hemicellulose content (HEC, %), fiber length (FL, mm), fiber width (FW, μm), and microfibril angle (MFA, °). The three growth traits were as follow: diameter at breast height (DBH, cm), tree height (H, m), and stem volume (V, m^3^). Relevant measurement methods of the phenotypic data for the 435 genotypes were described in previous study (Du et al., 2014).

### Discovery and Genotyping of SNP

The 435 unrelated individuals were resequenced to raw data (>15× coverage) by using the sequencing platform Illumina GA II. Then filtered reads were mapped to the genome of *P*. *trichocarpa* (a reference genome) by using SOAPaligner.2.20 with default value (Gu et al., 2013). The depth of effective mapping was ∼11× for most individuals and the rate of mapping in different accessions varied from 81 to 92%.

In order to acquire high quality SNPs, we uniquely used mapped paired end reads to execute SNP calling process. We calculated the genotype likelihood parameter of the genomic site for each individual by using SOAPsnp software of default parameters (Gu et al., 2013). To test the accuracy of our results of SNP calling, we compared it with our previous study of SNP data within 10 candidate genes of 120 individuals from genome re-sequencing, and that data was calculated by using PCR-Sanger sequencing (Du et al., 2014) randomly. The relative accuracy rate of SNP calling was more than 99%, indicating the high quality of platform of the SNP calling. According to the annotation of the *P. trichocarpa* genome (version.3.0) which were downloaded from Phytozome database, the gene-derived double-alleles SNPs in the full-length genes, including 1 kb downstream and 2 kb upstream of this genes, were extracted by using VCFtools (Petr et al., 2011)^8^ (Goodstein et al., 2012).

## Data Analysis

### Analysis of Single SNP-Based Association

We used TASSEL.v.5.0 (Bradbury et al., 2007) with mixed linear model (MLM) for exploring single nucleotide polymorphism (SNP)-based association in the association population of *Populus tomentosa*. The formula of MLM was as follow: y = μ + e + Qv + Zu, with μ indicating the intercepts vector, e indicating random experimental error, e indicating error of random experimental, Q matrixes indicating the structure of population, v indicating a vector for population effects, and Z being the matrixes relating y to u.

Furthermore, the pairwise kinship (K) and estimated membership probability (Q) were used to assess the population structure and relatedness among marker trait associations of individuals. We assessed the K matrix with previous methods (Grynko and Shkuratov, 2003; Evanno et al., 2010) and the Q matrix was assessed depending on subpopulations (*k* = 3) based on the previous related analysis (Evanno et al., 2010) by STRUCTURE V.2.3.4 (Evanno et al., 2010; Nick et al., 2013). Finally, we used positive false discovery rate (FDR) to make a correcting multiple test by employing the *Q* value package in R (Reiss et al., 2012).

### Analysis of Haplotype-Based Association

We conducted haplotype trend regression analysis to assess the haplotype frequencies from the genotype data, and estimate the potential haplotype-based associations (Zaykin et al., 2002; Gad and Ron, 2005). Based on 104 permutation tests in our study, the significance of singleton alleles and the frequencies of haplotype-based associations which lower than 0.05 were abandoned in this analysis.

## Results

### Identification of *FRK* Genes in 20 Plant Species

To perform a comprehensive analysis of *FRK* gene family in plant species, we firstly performed an analysis of phylogeny on the *FRK* genes of 20 plant species (Phytozome.v11.0, see text footnote 9), including six chlorophytes: *C. reinhardtii*, *D. salina*, *V. carteri*, *C. subellipsoidea*, *M*. *pusill* and *Micromonas* sp. RCC299; two embryophytes: *M. polymorph*a and *P. patens*; one lycophyte: *Selaginella moellendorffii*; one gymnosperms: *Picea abies*; one basal angiosperm: *A. trichopoda*; eight rosids: *B. distachyon*, *B. napus, A. thaliana*, *S. lycopersicum, E. salsugineum*, *V. vinifera, E. grandis* and *P. trichocarpa*; and two monocots: *Zea mays* and *Oryza sativa*. *FRK* genes of *A. thaliana* (Riggs et al., 2017; Supplementary Dataset S1) were used as reference sequences to identify *FRK* genes from the Phytozome database using a BLASTp search, generating 143 putative *FRK* genes in total. Then the Pfam database was used to verify the presence of the PfkB domain and 18 genes were eliminated in this process. Thus, we identified 125 *FRK* genes across 20 diverse plant species (Supplementary Dataset S1), with the largest number of *FRK* genes in *P. abies* (a gymnosperm; 12) and the smallest number in *C. subellipsoidea* (green algae; 1). Notably, we found that 15 *FRK* genes were already present in green algae (Supplementary Dataset S1), suggesting that *FRK* genes appeared early in evolution.

We also calculated and analyzed the percentage (%) of all protein-coding genes represented by *FRK* genes and found that *FRK* genes account for 0.023% of all genes in *P. trichocarpa*, 0.018% in *S. moellendorffii* (a basal angiosperm), and 0.007% in *V. carteri* (a green algae). Interestingly, the percentages of *FRK* genes among all *P. trichocarpa* genes are 1.3–2.8 times more than the percentages of that in ancient plants. This may be the result of a *Populus* lineage-specific paleotetraploidization (Tuskan et al., 2006).

We analyzed the features of *FRK* genes and our results showed a high variability of genetic features across the 20 plant species (Supplementary Dataset S1). The *FRK* genes of the 20 species were characterized as having 2–19 exons, with the fewest exons detected in green algae. As shown in our dataset, the *FRK* genes in the 20 plant species encode FRK proteins ranging from 71 to 1,207 amino acids in length with the longest in *P. paten*s and the shortest in *P. abies.* The relative molecular mass varied from 16.12 kDa (green algae) to 68.98 kDa (*A. thaliana*) and the pIs ranged from 4.22 (*P. abies*) to 7.13 (*V. vinifera*) (Supplementary Dataset S1). Together, our phylogenetic analysis of the *FRK* genes indicates that an event of domain loss took place early in the evolution of algae, and before the evolution of land plant lineages.

### Extensive *FRK* Gene Structure Reorganization During the Transition to Land Plants

**FIGURE 1:**
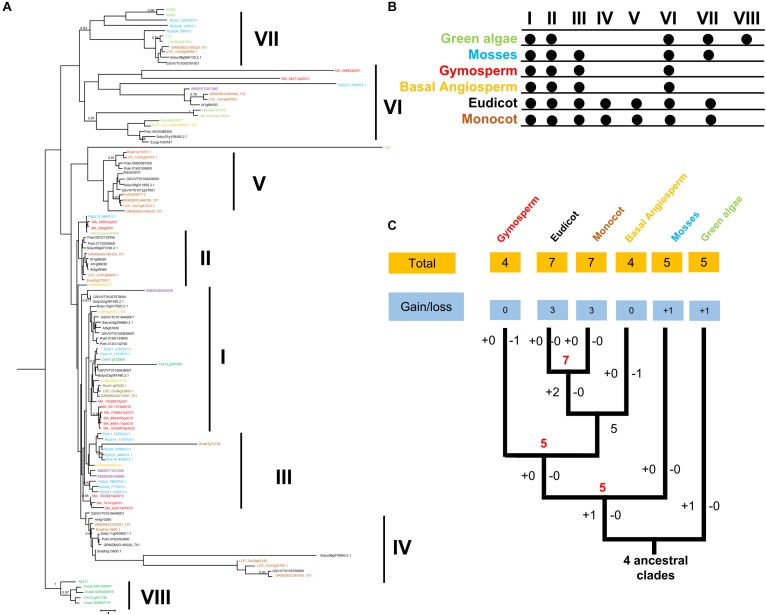
**(A)** Phylogeny of FRK proteins. Bootstrap values are shown; values < 0.80 were omitted. The scale bar indicates the number of changes per site. The tree was constructed using amino acid alignment. The eight clades of FRKs are indicated at the right of the tree. The tree is accompanied by a table to summarize the presence (∙) or absence (empty) of the clades in the plant family. **(B)** Birth and death clades of *FRK* families. The (+) on the branch represents the increase of clade and (–) represents the decrease of clade, and the number on the node represents the number of clade of ancestor species. **(C)** Plant divisions are color coded depending on their taxonomy classification: green algae (green), embryophytes (dark blue), basal angiosperms (dark yellow), gymnosperms (red), eudicot (black), and monocot (gray).

To examine the phylogenetic relationships among the land plants, we constructed a phylogenetic tree by using a maximum likelihood (ML) method (Figure 1). In addition, other methods, such as, neighbor-joining (NJ) method, were also used to reconstruct the phylogenetic tree of *FRK* family. As a result, the topology of NJ and ML trees were very similar. We also noticedthe topology of NJ and ML trees were very simila that the intron-exon structures supported the classification of *FRK* clades. All *FRK* genes were classified into eight clades (Figure 1). Each clade contained 5–20 members. Interestingly, five *FRK* clades (I, II, VI, VII, and VIII) were already present in green algae with high supporting values. Remarkably, clade VIII is specific to green algae with a high specific intron-exon structure and specific motif composition (Supplementary Figure S2).

In an embryophyte, an ancient land plant, five *FRK* clades (I, II, III, VI, and VII) were detected, suggesting that clade VIII was lost and clade III was gained before the divergence between green algae and embryophytes. In gymnosperms, basal angiosperms, and lycophytes, only four clades (I, II, III, and VI) were observed, suggesting that clade VII was lost during the transition (Figure 1). Notably, in the angiosperms including eudicot and monocot, we observed an extensive diversity of *FRK* genes with seven clades, including two angiosperm-specific clades (IV and V) (Figure 1). This may suggest that *FRK* genes of eudicot and monocot may have the same origin.

In general, the same motif composition was observed in the same clade, implying functional similarities in the same clades (Figure 1 and Supplementary Figure S2). However, we observed some clade-specific motifs across all the clades, which is highly consistent with topology of phylogenetic tree. For example, as shown in Figure 1, the LVXC motif, a sequence of 20 amino acid residues, was identified in the N-terminus of amino acid of FRKs, and it almost appeared in all the FRKs of different clades. In contrast, the LVXC motif-less lineage was only conserved in clade IV of angiosperm species (Supplementary Figure S2), indicating that clade IV may appear later in evolution. The ILK motif was conserved in all *FRK* of clade VIII, VII, and VI (Supplementary Figure S2), which indicated that the three clades may origin from the same ancestors. In this regard, there were most diverse motifs found in clade VIII (Supplementary Figure S2). Specially, PAFK motif appeared in clade VIII and was not found in any other clades (Supplementary Figure S2).

**FIGURE 2:**
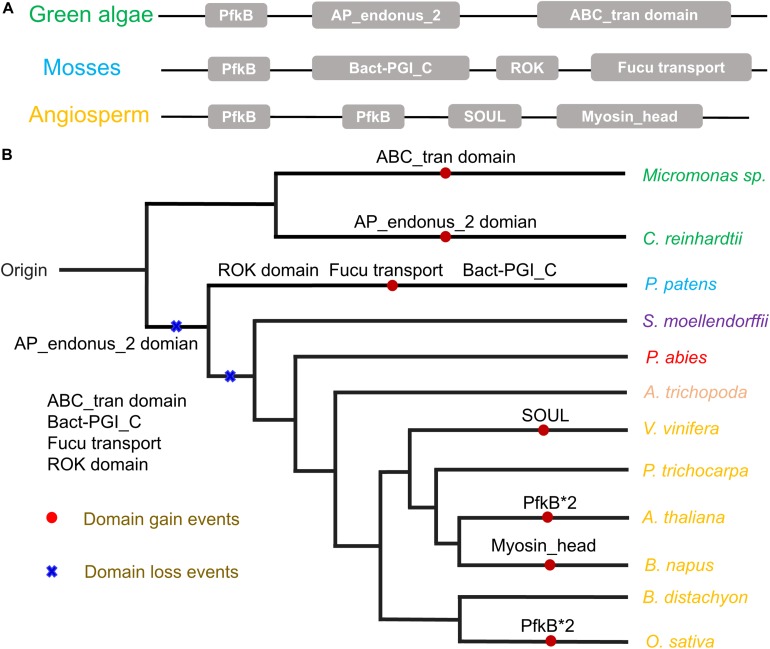
The auxiliary domain structure and the hypothetical evolutionary model in the 125 FRKs. **(A)** Domain structure of the FRK proteins found in green algae, mosses, and angiosperms. **(B)** Hypothetical evolutionary model for domain structure in the FRK proteins. Red circles represent domain gain events and blue crosses represent domain loss events inferred from the Pfam database. Plant divisions are color coded depending on their taxonomy classification: green algae (green), mosses (dark blue), lycophytes (violet), gymnosperms (red), basal angiosperms (flesh), and angiosperms (yellow).

The auxiliary domains in *FRKs* can be useful to track and analyze the potential evolutionary process and functional divergence of the *FRK* gene family, including gene loss and gene duplication events. We then explored the conservation and the evolution of auxiliary domains between the different protein clades. The AP_endonuc_2 domain (Xylose isomerase-like TIM barrel; PF01261) and ABC_tran domain (ATP-binding domain of ABC transporters; PF00005) were detected in *FRK* genes in the ancient clades VIII and VII (Figure 2 and Table 1). Following the parsimony rule, we inferred that the incorporation of these domains occurred in the early stage of plant species evolution, and before the green algae-land plant split (550 Mya). Likewise, the bact-PGI_C (Glucose-6-phosphate isomerase; PF10432), ROK (PF00480), FucU transport (PF05025), and ABC_tran domain (PF00005) were found in an ancient land plant, *P. patens* (Figure 2 and Table 1). Importantly, the ABC_tran domain was found to be conserved in the green algae and *P. patens*, indicating that the ABC (ATP-binding cassette) transporter domain may play essential roles in communication across cell membranes (Mccormick and Johnstone, 1990). This suggests that the AP_endonuc_2 domain was lost and two domains (the FucU transport domain and the ROK domain) were gained after the split of green algae and *P. patens* (Figure 2 and Table 1).

**TABLE 1:** Auxiliary domains in FRK proteins from different plants.

**Classes**	**Species**	**Auxiliary domains^a^**
Green algae	*C. reinhardtii*	ABC transporter, AP_endonuc_2
	*C. variabilis*	AP_endonuc_2
	*M. pusilla*	pfkB*2
Mosses	*P. patens*	ABC transporter, RbsD / FucU transport, bact-PGI_C, ROK
Angiosperms	*O. sativa*	pfkB*2
	*A. thaliana*	pfkB*2
	*B. napus*	Myosin_head
	*V. vinifera*	SOUL

*^*a*^The domains located outside of the PfkB domain.*

However, we did not identify an auxiliary domain which located outside of these conserved domains in *S. moellendorffii*, *P. abies*, and *A. trichopoda*. Interestingly, some of the auxiliary domains were found in angiosperm plants (Figure 2B). For example, the SOUL heme-binding domain (PF04832) was found in *V. vinifera*, and the Myosin_head (motor domain; PF00063) domain was found in *B. napus*. Also, a duplication of the PfkB domain (PfkB^∗^2 domain) event took place in *O. sativa* and *A. thaliana* (Figure 2B and Table 1). These results further support the idea that the diversification of special gene families occurred during the origin and rapid diversification of the angiosperms (Hileman et al., 2006). Most importantly, combined with the phylogenetic tree analysis, we found that auxiliary domains tend to appear in specific lineage clades (Figure 1). The result suggests that the ancient *FRK* genes may perform multiple physiological functions and have experienced a stepwise loss of domains during the transition to land plants, and a rapid diversification before the divergence of angiosperms.

### Functional Divergence of Paralogous *FRK* Pairs Derived From Segmental Duplication

Based on previous studies, duplication of genome has always been seen as a contributor to gene family expansion (Freeling, 2009). There are five types of duplication events: singleton, dispersed, proximal, tandem, and segmental duplication (Xu et al., 2018). In order to explore the type of *FRK* gene duplication that occurred in *Populus*, we used the *P. trichocarpa* genome as a reference to assess which type of duplications was the way to the expand *FRK* genes in *Populus*. Then we compared the number of *PtoFRK* genes originated from different mechanisms in *P. tomentosa.* This analysis suggested that 88.89% (8 of 9) of the *PtoFRK* genes originated via duplication events, including 6 from segmental duplication, and 2 from dispersed duplication (Table 2). To verify this result, we detected the chromosome distribution of *PtoFRKs* and found that the chromosome sites of 6 *PtoFRK* genes were consistent with the regions where the whole genome duplication took place (Tuskan et al., 2006). Furthermore, according to the *Populus* dilatancy parameter (λ = 9.1 × 10^–9^), it is estimated that 6 *FRK* genes that originated from segmental duplication may have been produced from 1 Mya, which suggests that they appeared recently in evolution. Furthermore, low Ka/Ks values (<0.3) were detected among the six *PtoFRK* genes, suggesting a strong purified selective pressure (Table 2). Our findings showed that the segmental duplication is the main and key driving mechanism for the increased members of the *FRK* gene family, and the newly originated *PtoFRK* genes were found to have originated over a short period of time.

**TABLE 2:** Paralogous *FRK* genes.

**LOCUS_1**	**LOCUS_2**	**Ka**	**Ks**	**Ka/Ks**
*PtoFRK2*	*PtoFRK4*	0.0365	0.2074	0.175988
*PtoFRK5*	*PtoFRK6*	0.0558	0.329	0.169605
*PtoFRK1*	*PtoFRK3*	0.0654	0.2247	0.291055

**FIGURE 3:**
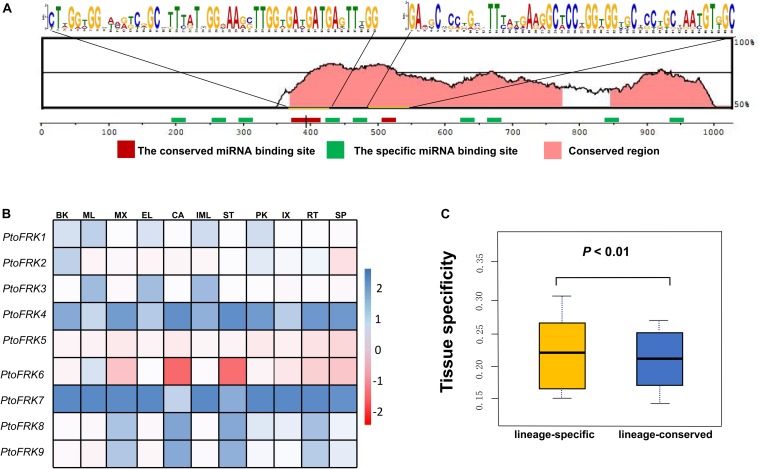
The miRNA target sites of *PtoFRK* genes. **(A)** mVISTA alignment of transcript sequences of *PtoFRKs*. The vertical bar indicates the sequence conservation level, the green rectangles indicate the binding site of specific miRNAs, and the red rectangles indicate the binding site of conserved miRNAs, and the conserved motifs were predicted by MEME. **(B)** RT-qPCR analysis of 9 *PtoFRKs* in 11 tissues in *P. tomentosa* including BK, bark; ML, mature leaf; MX, mature xylem; EL, elongated leaf; CA, cambium; IML, immature leaf; PK, leaf peak; IX, immature xylem; ST, phloem; RT, root; SP, stipe; the color scale represents the expression values. Each data point is the average of three biological replicates. **(C)** Comparison of tissue specificity of lineage-conserved *PtoFRKs* with lineage-specific *PtoFRKs.*

Consistent with gene balance hypothesis, previous studies showed that gene families of duplication-resistant can correspond to regulators or transcriptional factors that are overrepresented after events of whole genome duplication (WGD) (Freeling, 2009). We thus expected that salicoid duplicates, which were produced during whole-genome duplications in the lineage that produced the *Salix* (willow) and *Populus* genera, would be targeted by more *Populus*-specific miRNAs (Xie et al., 2017). As expected, all the *Populus FRK* genes were predicted to be regulated by *Populus*-specific miRNAs (miR7822, miR6421, miR6423, miR6448, miR6439, miR6452, and miR6462) and only two *FRK* genes are regulated by *Populus*-conserved miRNAs (miR171 and miR172) (Table 3). The result supported the idea that 90% of *Populus*-specific miRNAs target salicoid duplicates (Xie et al., 2017). Analysis of mVISTA alignment of *FRK* targets of miRNA revealed that cleavage sites of *Populus*-specific miRNAs are widely distributed in the range of 189 to 1,026 bp of transcript sequence and 25% of cleavage sites are located in non-conserved regions, while all of the cleavage sites of *Populus*-conserved miRNAs are located in highly conserved regions (Figure 3A and Table 3). Interestingly *PtoFRK1*, *PtoFRK3*, *PtoFRK2*, and *PtoFRK4* which derived from a segmental duplication are regulated by the same miRNA. However, *PtoFRK5* and *PtoFRK6* which derived from a segmental duplication are regulated by the different miRNA and the cleavage sites of miRNAs are different (Tables 2, 3). Consistent with this, they also have different transcription level in selected tissues (*P* < 0.01) (Figure 3). It infers that forced by segmental duplication in evolution, the regulating mechanism of gene-pairs may diverge.

**TABLE 3:** Potential regulation of *PtoFRKs* by miRNAs.

**Target gene**	**miRNA**	**Target site**	**miRNA**	**Target site**	**Effect**
*PtoFRK1*	miR7822	681–701	miR172	369–389	Cleavage
*PtoFRK2*	miR171	525–545	miR6421	409–429	Cleavage
*PtoFRK3*	miR6422	651–671	miR172	339–359	Cleavage
*PtoFRK4*	miR6421	467–487			Cleavage
*PtoFRK5*	miR6421	1106–1126			Cleavage
*PtoFRK6*	miR6423	284–395			Cleavage
*PtoFRK7*	miR6448	189–209	miR6439	927–946	Cleavage
*PtoFRK8*	miR6452	1086–1106			Cleavage
*PtoFRK9*	miR6462	1852–1871			Cleavage

*Target site position is given relative to the transcription start sit.*

Overall, the regulation patterns of FRK genes support the gene dosage balance hypothesis.

### Redundant and Divergent Patterns of *FRK* Expression During *Populus* Development and in Response to Stress

Previous reports showed that lineage-specific genes rapidly evolve and uniquely exist in plant lineages or individual species, and often perform unique biological functions (Yu et al., 2004). To further compare the functions between lineage-conserved and lineage-specific clades, we first analyzed the expression patterns of 9 *P. tomentosa* (*Pto*) *FRK* genes across various tissues, including mature leaf, immature leaf, elongated leaf, petiole, mature xylem, immature xylem, cambium, phloem, apex, and root. As a result, we found that most of the *PtoFRKs* showed a relative higher level in leaves tissues (Figure 3B), and that’s also consistent with a previous study which found that the *FRK* genes express highly in source tissue (Granot, 2007). Remarkably, most of the *PtoFRK* genes also showed a high expression level in xylem tissues, suggesting that FRKs may mainly function in wood formation. Then we contrasted the expression specificity of *Populus*-conserved *PtoFRK*s (*PtoFRK1*, *PtoFRK2*, *PtoFRK3*, *PtoFRK5*, and *PtoFRK6*) and *Populus*-specific *PtoFRK*s (*PtoFRK4*, *PtoFRK7*, *PtoFRK8*, and *PtoFRK9*). As a result, *Populus*-specific *PtoFRK* genes have lower expression levels and appear to be preferentially expressed in specific tissues (Figures 3B,C; *P* < 0.01), indicating a striking difference between *Populus*-conserved and *Populus*-specific *PtoFRK*s. In the same clades, *PtoFRK* genes showed a similar expression level and patterns, suggesting redundancy of function in the same clades. Overall, these observations suggest that newly originated *PtoFRK*s have high expression specificity, but generally lower expression levels.

To further examine the specificity of expression level of *PtoFRK* genes, we tested the expression of these genes under different conditions, such as treatment with the hormone ABA, or different stresses (drought, heavy metal, and high temperature). Indeed, some of these genes do show different expressions in response to distinct stress conditions (Supplementary Figure S1). Notably, *PtoFRK1* and *PtoFRK2* were differentially expressed in at least one of the four time points measured (1, 3, 6, or 12 h) under all abiotic stresses, suggesting that their functions may be biased toward more central roles in networks. However, under these stresses, no differences in expression level were detected in *PtoFRK*6, 7, 8, and 9 from lineage-specific clades.

### Lineage-Specific *PtoFRK* Genes Have Higher Potential for Functional Divergence

We used association analysis to further elucidate the potential functions of lineage-specific *PtoFRKs* in growth and wood formation. To this end, we have identified SNPs in the *PtoFRK* genes within the regions 1 kb downstream and 2 kb upstream of each gene. Totally, 4,446 common SNPs with frequency ≥ 0.05 within 7 full length of *PtoFRK* genes were detected, which approximate 19 SNPs per kb. The MLM module in TASSEL 5.0 was used to find significant associations between different phenotypes, including seven wood property traits and three growth (HC, holocellulose content; HEC, hemicellulose content; CC, cellulose content; LC, lignin content; FW, fiber width; FL, fiber length; and MFA, microfibril angle; H, tree height; V, stem volume; DBH, diameter at breast height), and genotypes for the SNPs, which were verified the accuracy with multiple testing by using FDR. We have identified 45 significant single marker associations (*P* < 0.01; *Q* ≤ 0.1) which represents 34 SNPs found in 7 candidate genes, and these associations would associate with two wood property and two growth traits. The individual SNPs explained between 10% (*PtoFRK3*-SNP24) and 22% (*PtoFRK6*-SNP87) (Supplementary Table S2) of the phenotypic variation, which was the highest contribution to phenotype, suggesting that the *PtoFRK* genes may play important roles in wood property traits and tree growth.

**FIGURE 4:**
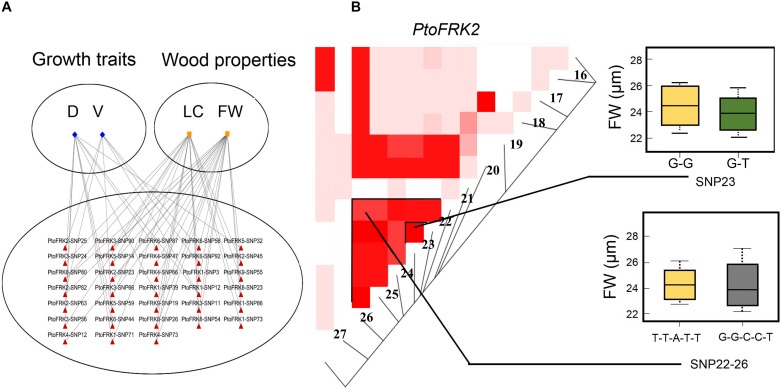
The single SNP-based associations and the haplotype-based associations in the association population of *P. tomentosa.* **(A)** The single SNP based associations between significant SNPs from *PtoFRKs* and their associated traits. The lines represent the significant associations. **(B)** The genotypic effect for the significant haplotypes of *PtoFRK*2-SNP22-26 and single SNP-based association of *PtoFRK*2-SNP23.

We contrasted the association of *PtoFRKs* of different tissues, which revealed a striking difference between *Populus*-conserved and *Populus*-specific *PtoFRK* genes. As expected, the marker-trait association analyses revealed that *Populus*-conserved *FRK* genes were more associated with growth and wood property traits. For *Populus*-conserved *FRKs*, 31 significant single-marker associations (*P* < 0.01, *Q* < 0.1), which represent 25 unique SNPs in *PtoFRK* genes, were detected, and they associated with four traits including two growth and two wood property traits. For example, 18 SNPs were significantly associated (*P* < 0.01, *Q* < 0.1) with D, FW, and V with 10.0% (*PtoFRK3*-SNP24) to 21.0% (*PtoFRK2*-SNP82) of the phenotypic variance (*R*^2^) (Figure 4A and Table 4). Eight SNPs (*PtoFRK5*-SNP14, *PtoFRK5*-SNP32, *PtoFRK5*-SNP59, *PtoFRK6*-SNP23, *PtoFRK6*-SNP44, *PtoFRK6*-SNP56, *PtoFRK6*-SNP87, and *PtoFRK6*-SNP92) associated with four traits (D, V, FW, and LC) with 20.0% (*PtoFRK5*-SNP14) to 22.0% (*PtoFRK6*-SNP87) of the phenotypic variance (Figure 4A and Table 4).

**TABLE 4:** Summary of significant SNPs within *PtoFRKs* associated with growth and wood properties in the association population of *P. tomentosa.*

**Gene**	**Number of associations^a^**	**Number of associated SNPs^b^**	**Traits**	**Range of *R*^2^** (%)
*PtoFRK1*	10	6	D, V, LC, FW	0.12–0.15
*PtoFRK2*	7	5	D, V, LC, FW	0.17–0.20
*PtoFRK3*	5	5	D, FW, LC	0.10–0.14
*PtoFRK4*	4	4	FW, LC	0.11–0.21
*PtoFRK5*	5	3	D, FW, LC	0.11–0.15
*PtoFRK6*	8	5	D, FW, LC	0.13–0.20
*PtoFRK8*	3	3	FW, LD	0.11–0.18
*PtoFRK9*	3	2	FW, LD	0.11–0.18

*^*a*^Number of associations: the number of associations detected (*P* < 0.01) in each *PtoFRK*. ^*b*^Number of associated SNPs: the number of associated SNPs detected for each *PtoFRK* (*P* < 0.01).*

Moreover, for lineage-specific *PtoFRK* genes, only 14 significant associations (*P* < 0.01, *Q* < 0.1) representing 9 unique SNPs in 4 *PtoFRK* genes were detected to be associated with two wood property traits (FW and LC). For example, four SNPs in *PtoFRK4* (*PtoFRK4*-SNP12, *PtoFRK4*-SNP47, *PtoFRK4*-SNP66, and *PtoFRK4*-SNP73) associated with FW and LC with 11.0% (*PtoFRK4*-SNP12) to 16.0% (*PtoFRK4*-SNP73) of the phenotypic variance (*R*^2^). In clade V, six SNPs in *PtoFRKs* (*PtoFRK8*-SNP26, *PtoFRK8*-SNP54, *PtoFRK8*-SNP80, *PtoFRK9*-SNP19, *PtoFRK9*-SNP32, and *PtoFRK9*-SNP55) associated with FW and LC with 12.0% (*PtoFRK8*-SNP80) to 13.0% (*PtoFRK8*-SNP54) of the phenotypic variance (Figure 4A and Table 4).

Furthermore, haplotype-based associations, performed by haplotype trend regression, were used to explore the genetic effect of *PtoFRKs*. Specially, one haplotype (T-T-A-T-T) from *PtoFRK2* associated with FW, which was supported by significant single SNP-based associations of *PtoFRK* genes with the same wood property traits (Figure 4B). This further supported the idea that this *FRK* can play an important role in wood formation (Figure 4B). Together, the significant marker-trait association analyses indicated that *FRK*, a core kinase in glycolysis, plays an important role in wood formation and growth in *P. tomentosa*.

## Discussion

### Domain Loss and Gain Events of the *FRK* Gene Family

To what we know today, radiation, and colonization of land plant are major or main keystones in the evolutionary process of living organisms (Graham et al., 2012), which can shape the diversity of plant species, landscape and atmosphere on Earth (Kenrick and Crane, 1997). This was accompanied by the changes including genome, physiological, and morphological to deal with the challenging conditions (Graham et al., 2012; Harholt et al., 2015). Therefore, the transition of plants from the oceans to land required several functional adaptations, and it must have been accompanied by multiple protein gain and loss events of motif or domain, for functional domains were the key elements of functional divergence, and they are the main components of proteins and are essential for protein function and protein interactions (Bagowski et al., 2010).

During wood formation, fructose is always phosphorylated to fructose-6-phosphate (F6P) by a fructokinase (FRK) in the beginning, which is necessary for sucrose-derived fructose metabolism because it facilitates carbon allocation to wood cell walls (Melissa et al., 2012). Here, by using publicly available genome databases, we first analyzed the phylogenetic relationships of *FRK* genes in 6 green algae and 14 land plants. The AP_endonuc_2 domain (PF01261) and ABC_tran domain (ATP-binding domain of ABC transporters; PF00005) were found to be present in green algae. Likewise, four auxiliary domains bact-PGI_C (PF10432), ROK (PF00480), FucU transport (PF05025), and ABC_tran domain (PF00005) were found in the moss *P. patens.* Several auxiliary domains were also detected in angiosperms. We thus speculate that auxiliary domain gain and loss during plant transition to land always played a key and indispensable role in both the functions of divergence of plant colonization and lineages. Moreover, the motif composition of different clades indicates that gain and loss of domain in evolution is a gradual process.

The origin of a new gene family is mainly conferred by the fusion or fission of protein domains in early streptophytes (Margulis, 1992; Simionato et al., 2007). The domain structure of proteins is crucial for their function; thus, the alteration of auxiliary domains is presumed to be related to the alteration of the functions of FRKs in biological processes and environmental adaptation of plants (Romani et al., 2018). Interestingly, some domains of FRK proteins were only found in green algae and moss, for example, the ABC transporter domain (PF00005), a water-soluble domain of transmembrane ABC transporters, which are often involved in the transmembrane transport of various molecules (the PDR-type ABC transporter strongly mediates cellular uptake of the phytohormone ABA; the ABC transporter (AtABCG25) is always involved in ABA responses and transport) (Danuta et al., 2003). In angiosperms, the Myosin_head domain (PF00063), which has been found in *B. napus*, mainly associates with converting chemical energy, in the form of ATP, to mechanical energy (Kawakubo et al., 2007). To verify our speculative history of evolution (Figure 2), *FRK* genes of some other plant which were not often used to evolutionary research have been provided in Supplementary Dataset S3, and we did not find auxiliary domians of *FRK* protein in other plant species (Figure 2).

Thus, it is likely that these auxiliary domains have the potential to contribute to the evolution of phenotypes and harbor many potential innovations essential for terrestrial life.

### Functional and Regulating Redundancy of *FRK* Genes in *Populus*

In contrast to green algae, functional redundancy of gene families is a ubiquitous phenomenon in land plants (Pérez-Pérez et al., 2013). Consistent with a previous study, there are only two members of the *FRK* gene family on average in green algae, which suggested that almost no duplication events occurred. By contrast, three whole-genome duplication (WGD) events were found during *Populus* evolution, which resulted in overrepresentation of genes associated with disease resistance, metabolite transport, and lignocellulosic wall biosynthesis (Tuskan et al., 2006). In our study, we have identified nine *PtoFRK* genes in the genome of *P. tomentosa*, whereas there are 12 *FRK* genes in *A. thaliana*. The most common domains occur in *P. tomentosa* compared with *A. thaliana* in a ratio ranging from 1.4:1 to 1.8:1 (Tuskan et al., 2006). The low ratio of *FRK* genes in *P. tomentosa* compared with *A. thaliana* might point to a stepwise reduction in *FRK* genes.

Based on the previous studies, segmental duplication and tandem duplication always played a key role in the expansion of *FRK*s in *A. thaliana*. Here, detailed analyses revealed that segmental duplication was the main mechanism of *PtoFRK* gene expansion in *P. tomentosa*, as we did not find any tandem duplication of *PtoFRK* gene clusters in the *P. tomentosa* genome. Phylogenetic analyses revealed that three lineage-conserved clades and two lineage-specific clades are present in *P. tomentosa*. Among them, 6 *PtoFRK* genes are apparently paralogous, suggesting functional redundancy of *FRK* genes in *P. tomentosa*. Supporting this, mostly *PtoFRK*s from the same clade had similar expression patterns, suggesting potential functional redundancy of *FRK* genes within the same clade. Also, most of *PtoFRK* paralogous pairs in *P. tomentosa* are regulated by the same *PtomiRNAs*, and the target site of regulating miRNA alignment also exhibited an obvious conservation structure of the canonical –G–(6X)–G– repeats, suggesting a possible functional redundancy between the recently duplicated *PtoFRK* gene pairs. Interestingly, a gene-pair (*PtoFRK5* and *PtoFRK6)* is regulated by the different miRNA and the cleavage sites of miRNAs are different (Tables 2, 3). It infers that regulating mechanism of gene-pairs may also be forced by segmental duplication in evolution.

### Functional Novelty of Lineage-Specific Gene Clades

After duplication, one gene copy often accumulates mutations, which can probably lead to functional divergence (Liu et al., 2018). One copy within a paralogous pair which originated from segmental duplication may often evolve in different mechanisms, such as neofunctionalization, subfunctionalization (Lynch and Force, 2000), or non-functionalization (Ohno, 1971). In our study, *FRK* genes of clades VIII, VII, and IV have been lost in the embryophyte, gymnosperm, and lycophyte genomes, respectively, and two lineage-specific clades (IV and V) emerged in angiosperms. It remains unknown today what the functional contribution of these different clades is to the evolutionary history and process. It is often assumed that mutations in evolutionarily conserved genes are mostly to be deleterious, therefore genes of lineage-conserved clades are biased toward more central roles in biological networks (Konstantin et al., 2007; Gershoni and Pietrokovski, 2014). In our study, we detected significant associations in *PtoFRK*s of *Populus*-conserved clades with four wood property and growth traits in trees. Moreover, the low tissue expression specificity and high expression level indicate that *FRK* genes of lineage-conserved clades often underwent strong selection pressure during evolution (Gramer and Huber, 1991; Plange et al., 2014).

Furthermore, highly conserved functional domains in lineage-conserved genes are often the targets of conserved miRNAs, which also play central roles in biological networks. By contrast, lineage-specific genes are often targeted by many newly originated miRNAs in a random way, and they often appear and choose to be expressed in a specific tissue in a preferential and special way, and are associated with less lineage-specific phenotypes. Therefore, lineage-specific genes have a greater chance to contribute to phenotypic variations because their roles are not absolutely essential. Furthermore, auxiliary domains and specific motifs are preferentially found in lineage-specific genes, suggesting functional differentiation and novelty of lineage-specific gene clades. Thus, the expansion of *FRK* genes seems to be associated with phenotypic changes and lineage-specific processes that shape the development of plants. Our study extends the exploration of more specific roles of members of large gene families throughout the plant genome, and highlights the important and key selective pressures which can influence regulatory networks.

## Conclusion

In summary, we explored hypothetical evolutionary pathways of *FRK* gene family in 20 plant species and the regulating mechanism and functional divergence in *Populus*. Totally, 125 putative *FRK* genes can be organized into 8 clades, including 3 conserved clades and 5 specific clades. Evolutionary analysis revealed that *FRK* genes transient to vascular plants, accompanied by loss, acquisition and diversification of functional domains. In *Populus*, segmental duplication often appears to be the major mechanisms of the expansion of *FRK* genes, and most *FRK* genes that were duplicated in salicoids can be regulated by *Populus*-specific miRNAs. Furthermore, we detected the *FRK* gene family expanded accompanying lineage-specific traits and they often experienced multiple duplication events which help to promote fundamental neofunctionalizations and subfunctionalizations in evolution. All in all, our results contribute to explore more specific roles of members in gene families throughout the genome and provide a new framework for further functional investigation of gene family.

## Accession Numbers

The raw data of genome resequencing have been deposited in the Genome Sequence Archive in BIG Data Center (BIG, CAS, China) under accession number of CRA000903.

## Data Availability Statement

The variation data of this manuscript have been deposited in Big Data Center, Beijing Institute of Genomics (BIG), Chinese Academy of Sciences, under the accession number GVM000056 that are publicly accessible at https://bigd.big.ac.cn/gvm/getProjectFile?t=56b6b35f.

## Author Contributions

DZ and JX designed the experiment and conception, and obtained the funding and were responsible for this manuscript. WX tested the experiments and wrote the manuscript. YZ helped to analyze and assess the data. JX and SC provided the valuable suggestions on the manuscript. JX, SC, YZ, and DZ helped to revise the manuscript. All authors read and approved the manuscript.

## Conflict of Interest

The authors declare that the research was conducted in the absence of any commercial or financial relationships that could be construed as a potential conflict of interest.

## Footnotes

1 http://www.phytozome.net/

2 http://pfam.xfam.org/

3 https://web.expasy.org/protparam/

4 http://meme-suite.org/tools/meme

5 http://www.ebi.ac.uk/Tools/pfa/iprscan/

6 http://gsds.cbi.pku.edu.cn/

7 http://plantgrn.noble.org/psRNATarget/

8 https://phytozome.jgi.doe.gov/pz/portal.html
