# Supplementary material for: Evolution and Functional Divergence of the Fructokinase Gene Family in Populus
Source: Front Plant Sci. 2020 May 14;11:484. doi: 10.3389/fpls.2020.00484 (PMC7243158; doi:10.3389/fpls.2020.00484)

Supporting_data

Table S1. The primers used for RT-qPCR in this study.

Figure S1. The expression levels of 9 *PtoFRKs* were analyzed by RT-qPCR under 4 abiotic stresses including ABA, drought, heavy metal, and high temperature at five time points including 0, 1, 3, 6, and 12 h. Gene expression levels were normalized with transcript values. Each data point is the average of three biological replicates.

Figure S2. Motifs divisions are color coded based on their sequence classification: YDPN-motif (red), di-gly(GG)-motif (dark green), ARTA-motif (yellow), SELB-motif (green), G/AXGD-motif (brown), LVXC-motif (violet), ILK-motif (orange) and PAFK-motif (blue).

Data sets

Supplementary Data 1. List of the domains, length of amino acid sequences, pIs, and molecular mass of plant *FRK* genes used in this study.

Supplementary Data 2. Details of significant SNPs within *PtoFRKs* associated with wood property and growth traits in an association population of *P. tomentosa*.

Supplementary Data 3. List of plant *FRK* genes used to find auxiliary domains.

Table S1 The primers used for RT-qPCR in this study.

Gene	Forward Primer	Reverse primer
PtoFRK1	GAAAACGGCGTGATCGCTAC	CCTTGGCTCATCCTCAAGCA
PtoFRK2	GACGTGATCGCTTCCGGTAT	ACTCTTCGGGCCTTAGCAAC
PtoFRK3	GAAAACGGCGTGATCGCTAC	CCTTGGCTCATCCTCAAGCA
PtoFRK4	GGCCGGAAAAAGAAACCCAC	CACCGTCCAACAGTTCCGTA
PtoFRK5	AGCAATTGGAAATGTGTAGGTGA	TGCAGGGATAGCACCTCTCT
PtoFRK6	CCCAGGGAAGAGATGGGAGA	ATTTCTGGCGGTGGCATGTA
PtoFRK7	GGTCAAGCCATATTGCAGCG	GTAGTGCAGGGATTGCACCT
PtoFRK8	TGCAAATCAGCCCACATTGC	AGCTGGAATTGCACCTCTCC
PtoFRK9	TCTGGCATGATCGCCTCAAG	TCTTCCTTTGAAAAGTGCCTTAGT

Figure S1. The expression levels of 9 *PtoFRKs* were analyzed by RT-qPCR under 4 abiotic stresses including ABA, drought, heavy metal, and high temperature at five time points including 0, 1, 3, 6, and 12 h. Gene expression levels were normalized with transcript values. Each data point is the average of three biological replicates.

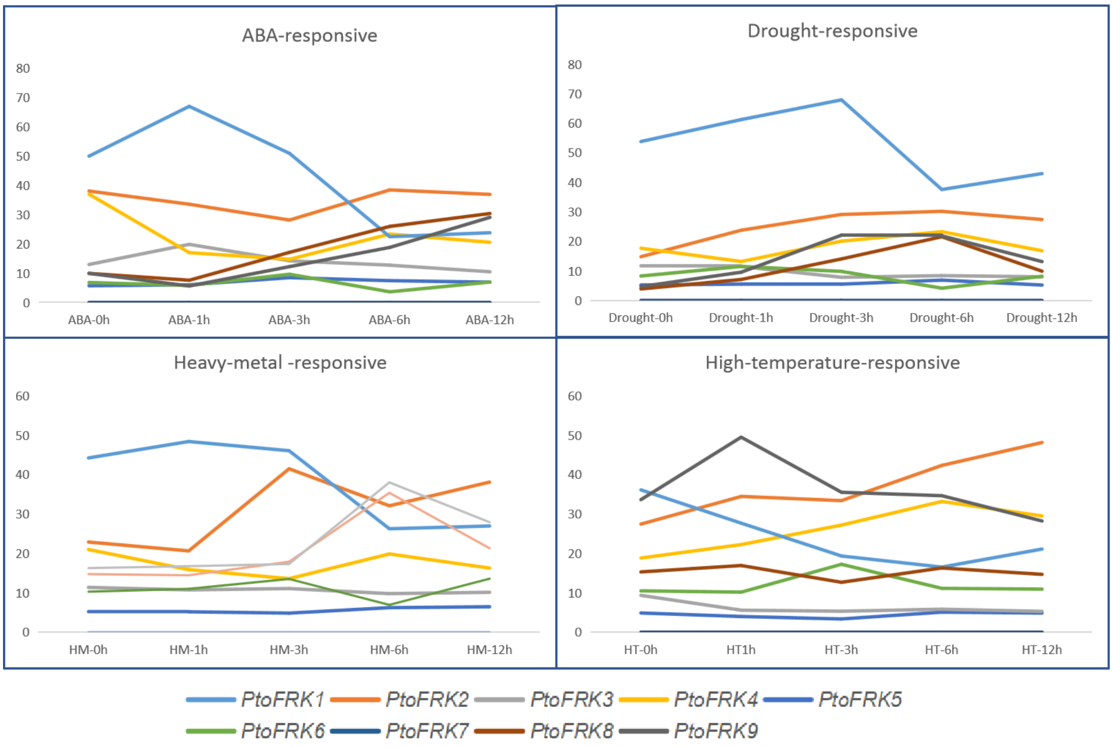

Figure S2. Motifs divisions are color coded based on their sequence classification: YDPN-motif (red), di-gly(GG)-motif (dark green), ARTA-motif (yellow), SELB-motif (green), G/AXGD-motif (brown), LVXC-motif (violet), ILK-motif (orange) and PAFK-motif (blue).

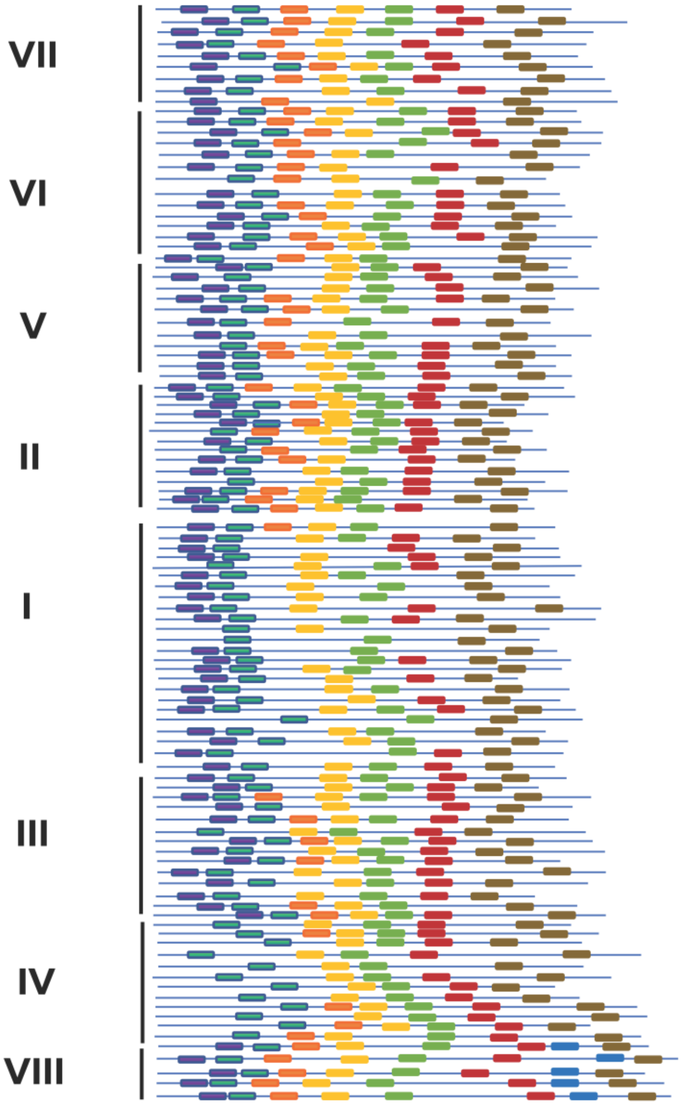

Supplement: Supplementary file 1 [file Presentation_1.pdf]
